# Human blood serum can donor-specifically antagonize effects of EGFR-targeted drugs on squamous carcinoma cell growth

**DOI:** 10.1016/j.heliyon.2021.e06394

**Published:** 2021-03-11

**Authors:** Dmitry Kamashev, Maksim Sorokin, Irina Kochergina, Aleksey Drobyshev, Uliana Vladimirova, Marianna Zolotovskaia, Igor Vorotnikov, Nina Shaban, Mikhail Raevskiy, Denis Kuzmin, Anton Buzdin

**Affiliations:** a Shemyakin-Ovchinnikov Institute of Bioorganic Chemistry, Russian Academy of Sciences, 16/10, Miklukho-Maklaya St., Moscow 117997, Russia; b World-Class Research Center "Digital Biodesign and Personalized Healthcare", Sechenov First Moscow State Medical University, 8-2, Trubetskaya St., Moscow 119992, Russia; c Moscow Institute of Physics and Technology (National Research University), Moscow Region 141700, Russia; d Blokhin National Medical Research Center of Oncology of the Ministry of Health of Russia; e OmicsWay Corp., Walnut, CA, USA

**Keywords:** Lung cancer, Colorectal cancer, EGFR, HER-targeted therapy, Human blood serum, Cetuximab, Erlotinib, Trastuzumab, EGF, Neuregulin, NRG, TGF-alpha, Squamous cell carcinoma, A431, Drug resistance

## Abstract

Many patients fail to respond to EGFR-targeted therapeutics, and personalized diagnostics is needed to identify putative responders. We investigated 1630 colorectal and lung squamous carcinomas and 1357 normal lung and colon samples and observed huge variation in EGFR pathway activation in both cancerous and healthy tissues, irrespectively on *EGFR* gene mutation status. We investigated whether human blood serum can affect squamous carcinoma cell growth and EGFR drug response. We demonstrate that human serum antagonizes the effects of EGFR-targeted drugs erlotinib and cetuximab on A431 squamous carcinoma cells by increasing IC50 by about 2- and 20-fold, respectively. The effects on clonogenicity varied significantly across the individual serum samples in every experiment, with up to 100% differences. EGF concentration could explain many effects of blood serum samples, and EGFR ligands-depleted serum showed lesser effect on drug sensitivity.

## 1 Introduction

The HER receptor family consists of four receptor tyrosine kinases. In humans, these include EGFR (HER1), HER2 (Neu), HER3, and HER4 proteins, encoded by the *EGFR, ERBB2, ERBB3*, and *ERBB4* genes, respectively [1, 2]. Several growth factors, called HER ligands, are known to have the capability to bind these receptors and activate them [3, 4]. Inactive HER receptors exist as the monomers spanning the plasma membrane. When HER ligands bind the extracellular domains (ectodomains) of EGFR, HER3, or HER4, the monomeric receptors homo- or heterodimerize and become functionally active through reciprocal intramolecular tyrosine phosphorylation of their kinase domains [5]. EGFR molecules are essential for the mediation of both proliferative and survival signals to cells [6, 7]. Overexpression of the EGFR receptors, as well as their hyperactivating mutations, play a major role in proliferative signaling in a variety of cancers [5, 8, 9]. A therapeutic approach has been developed to block EGFR receptor activities by targeted drugs specifically: the use of monoclonal antibodies (mAbs) including cetuximab, or receptor tyrosine kinase inhibitors such as erlotinib in EGFR targeting for the treatment of carcinoma cell subtypes of head and neck, colorectal, pancreatic, and lung cancers has been clinically validated [10, 11, 12, 13].

Although the overall response rate to EGFR inhibitors can be as high as 66% [14], the specific predictors of clinical response are of great practical importance. These are: a history of smoking, results of protein mass spectra analysis of blood serum samples [15], gene expression-based molecular pathway activation features [16, 17], EGFR expression level, and, most importantly, activating mutations of *EGFR* and downstream regulatory kinase genes such as *KRAS, NRAS* and *BRAF* [18, 19]. Other possible predictors of clinical response to EGFR inhibitors have not been validated yet.

Several groups previously reported the rescuing effects of EGFR ligands (EGF, NRG) on the viabilities of cells treated with EGFR-targeting drugs [20]. Thus, tumor response to the HER-targeted therapy can be affected by a variety of extracellular factors present in the patient body, particularly in human peripheral blood. These factors modulate the anti-proliferative action of the drugs during the patients' treatment.

Here, we investigated *in silico* activities of EGFR pathways in 604 colorectal and 1026 lung squamous carcinoma samples and compared them with the activities in 578 normal lung and 779 normal colon samples. We observed significant variation in EGFR signaling activation in both cancerous and normal tissues. Although *EGFR*-mutated lung cancers showed greater activation profiles (*p* < 0.001), the wild-type tumors also had a high variation that could not be explained by the tumor's intrinsic properties. Therefore, we investigated whether external factors like blood serum can affect squamous carcinoma cell growth and response to EGFR-targeted drugs. On A431 squamous carcinoma cells that overexpress EGFR [21], we show that peripheral blood serum samples, which we took from seven healthy female donors, could antagonize the effects of EGFR-targeted drugs erlotinib and cetuximab by increasing their half-inhibitory concentrations by about 2- and 20-fold, respectively. The effects on clonogenicity varied statistically significantly across the individual serum samples in every experiment, with differences of up to 100%. We show that serum devoid of EGFR ligands exerted significantly lower effects on sensitivity to erlotinib. We measured concentrations of EGFR ligands EGF, TGF alpha, and NRG, and found that EGF in a physiological concentration range can explain most of the effects observed here for the blood serum samples. Our findings suggest that profiling EGFR ligands in the patient's blood can be a further step towards the personalization of treatment of squamous carcinomas with EGFR-targeted drugs.

## 2 Results

### 2.1 EGF pathway activation level varies significantly in both cancerous and normal tissues

First, we compared the activation level of the EGFR pathway across normal and cancerous human lung and colon tissues because these are the primary localizations in EGFR-targeted therapies (Table 1). RNA sequencing gene expression profiles of 578 normal lung and 779 normal colon samples were extracted from the Genotype-Tissue Expression (GTEx) database [28]. In turn, 604 colorectal (CR) and 1026 non-small-cell lung cancer (NSCLC) RNA sequencing expression profiles were extracted from The Cancer Genome Atlas (TCGA) database [29]. Pathway activation levels (PAL) were calculated for two versions of the EGFR pathway: one extracted from Biocarta [30] and another from Qiagen Pathway Central [31] databases.

**Table 1:** Approved indications for the use of EGFR inhibitors in clinical oncology.

Drug generic name	Approved cancer types	Companion biomarkers	Reference
Cetuximab	Colorectal cancer, head and neck cancer	Wild-type *KRAS, NRAS, BRAF* (for colorectal cancer);	[22, 23]
Panitumumab	Colorectal cancer	Wild-type *KRAS, NRAS, BRAF*	[22]
Necitumumab	Untreated squamous non-small cell lung cancer (NSCLC)	NA	[24]
Afatinib	NSCLC, breast cancer	Activating mutations in *EGFR* (for NSCLC): exon 19 deletions or exon 21 L858R mutation (for NSCLC)	[25]
Gefitinib	NSCLC	Activating mutations in *EGFR*: exon 19 deletions or exon 21 L858R mutation	[26]
Erlotinib	NSCLC, pancreatic cancer	Activating mutations in *EGFR*: exon 19 deletions or exon 21 L858R mutation (for NSCLC)	[25]
Dacomitinib	NSCLC	Activating mutations in *EGFR*: exon 19 deletions or exon 21 L858R mutation	[25]
Osimertinib	NSCLC	Activating mutations in *EGFR*: exon 19 deletions or exon 21 L858R mutation, plus T790M mutation; but no exon 20 C797S mutation	[27]

To calculate PAL, we used the Oncobox method that quantifies pathway activation using whole-transcriptome data and the knowledge of an activator/inhibitor function of each pathway component [32].

This method of PAL calculation was shown to suppress the batch effects in various series of gene expression measurement experiments [33], and to minimize the errors introduced by the high-throughput methods of transcriptome analysis [34, 35]. PAL's absolute value reflects the strength of a pathway perturbation, and a positive or negative value indicates pathway activation or downregulation, respectively [32, 36].

**Figure 1:**
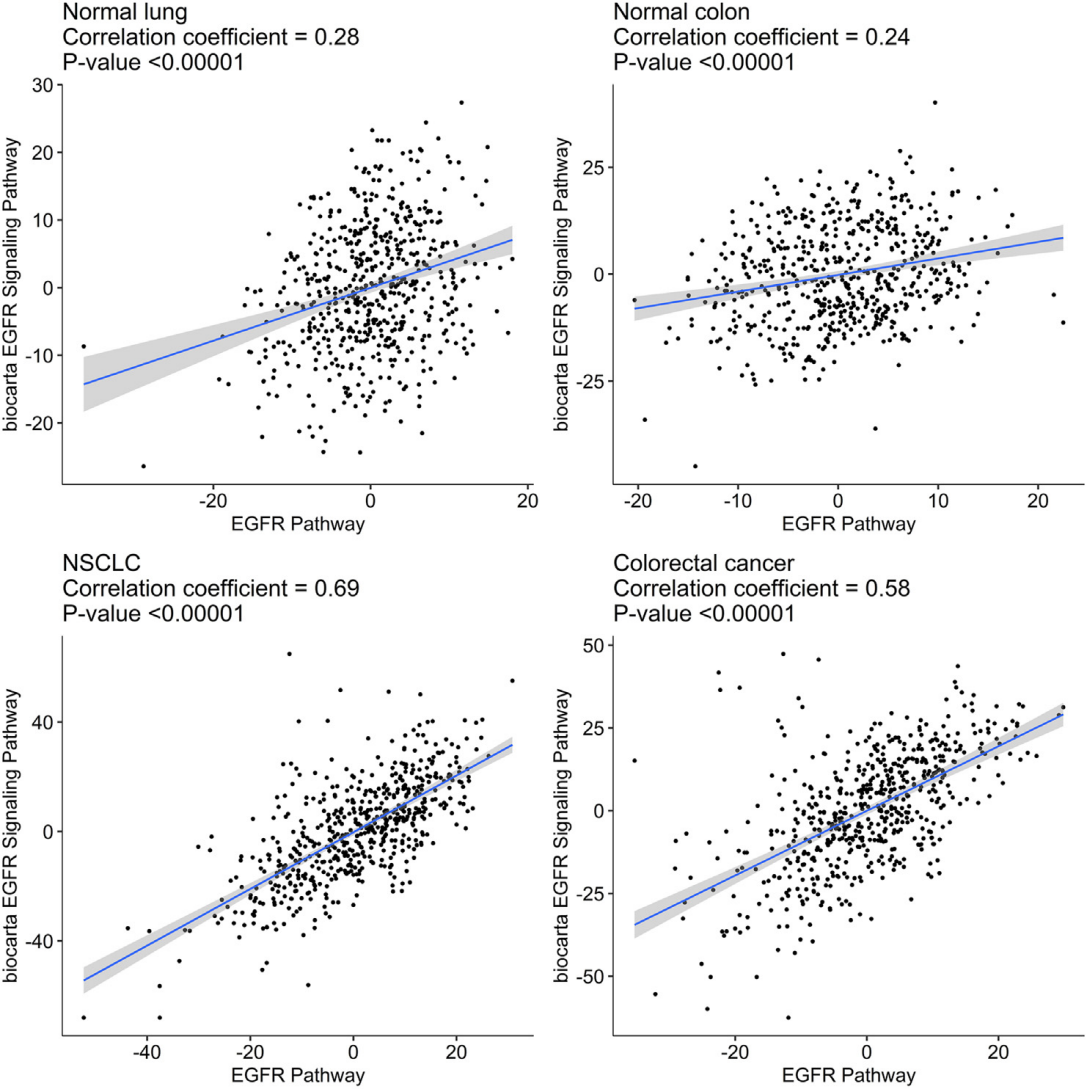
Correlation of pathway activation level (PAL) of the EGFR pathway from *Qiagen Pathway Central* annotation with PAL of the EGFR pathway from *Biocarta pathway annotation.* Panels represent correlations for the cancerous (TCGA database) and normal (GTEx database) lung and colon tissues.

**Figure 2:**
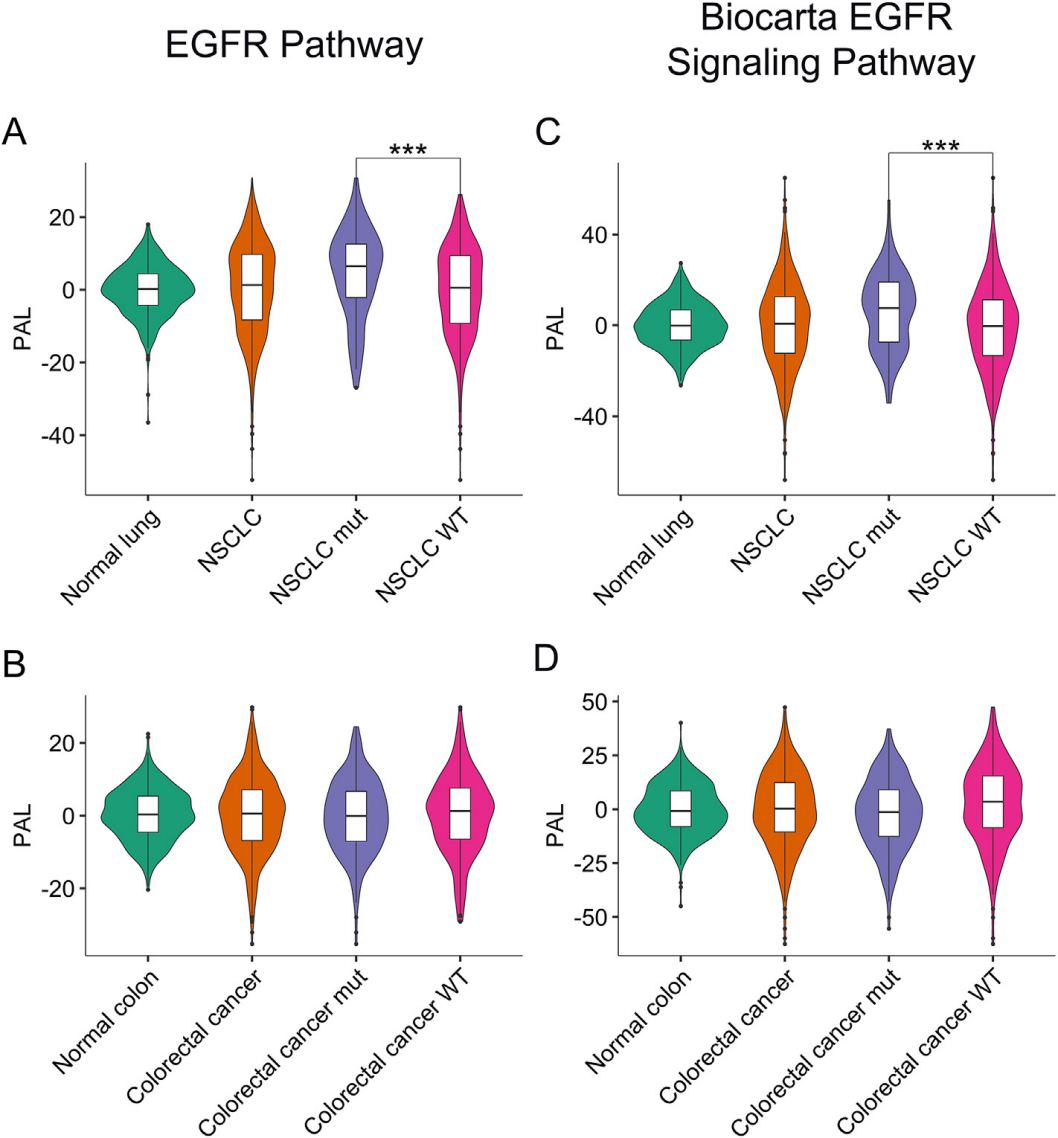
Distributions of pathway activation level (PAL) for the EGFR pathway in cancerous and normal tissues. A-B, distributions for the EGFR pathway (*Qiagen Pathway Central)* for lung (A) and colon (B) tissues. C-D, distributions for the EGFR pathway (*Biocarta*) for lung (A) and colon (B) tissues. Cancerous samples were also subdivided into wild type samples (WT) and samples harboring mutations in *EGFR, KRAS, NRAS,* or *BRAF* genes (mut).

We analyzed two versions of the EGFR pathway because they have somewhat different gene compositions, which can influence the results. However, we observed statistically significant correlations between the PAL values of both EGFR pathway versions (*p* < 0.00001), Figure 1. For both versions, we found that the activation levels of the EGFR pathway varied significantly in both the cancerous and normal tissues interrogated (Figure 2). In addition, we subdivided the cancer samples into two groups: (i) tumors that had known clinically relevant mutations in EGFR signaling genes: *EGFR, KRAS*, *NRAS,* and *BRAF*, and (ii) “wild type” tumors that did not have these mutations. In the “mutated” NSCLC cancers, we observed statistically significantly greater EGFR pathway activation levels compared with the “wild-type” cancers (*p* < 0.001, Figure 2A, C). The same was not the case for the CR cancer samples, which showed indistinguishable activation trends for both “mutated” and “wild-type” tumors (Figure 2B, D). However, the intragroup standard deviations were not statistically significantly different in the “mutated” and “wild-type” tumors in both NSCLC and CR cancers (Figure 2).

**Figure S1:**
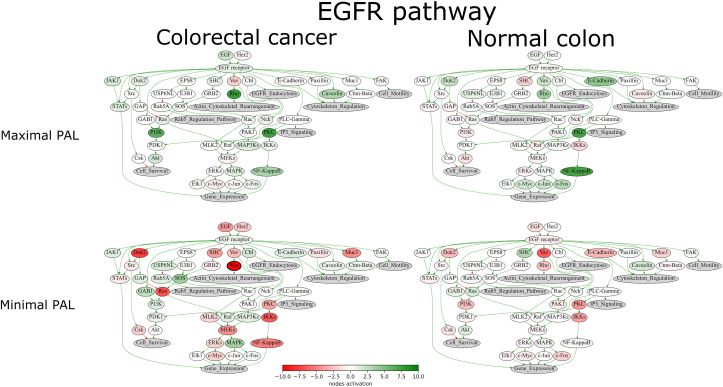
EGFR pathway (*Qiagen Pathway Central*) shown as an interacting network for samples with maximal (top) or minimal (bottom) PAL in colorectal cancer (left) vs normal colon (right). Green/red arrows indicate activation/inhibition interactions, respectively. The color depth of transcript nodes reflects the extent of node activation (logarithms of the expression fold change for each node, the reference is the geometric average between expression levels in all samples in the respective groups). Visualized using Oncobox software.

**Figure S2:**
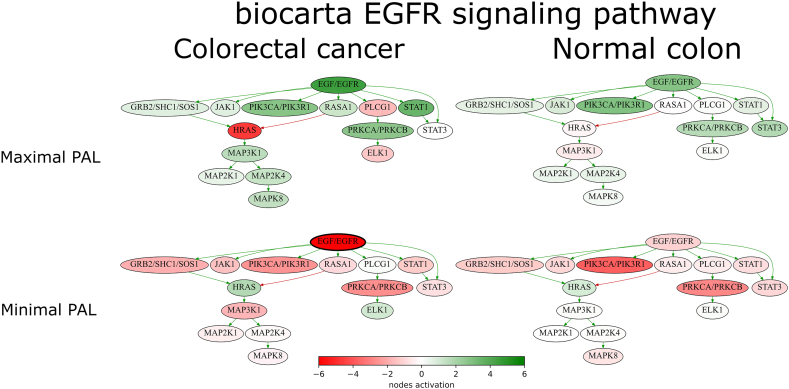
EGFR pathway (*Biocarta*) shown as an interacting network for samples with maximal (top) or minimal (bottom) PAL in colorectal cancer (left) vs normal colon (right). Green/red arrows indicate activation/inhibition interactions, respectively. The color depth of transcript nodes reflects the extent of node activation (logarithms of the expression fold change for each node, the reference is the geometric average between expression levels in all samples in the respective groups). Visualized using Oncobox software.

**Figure S3:**
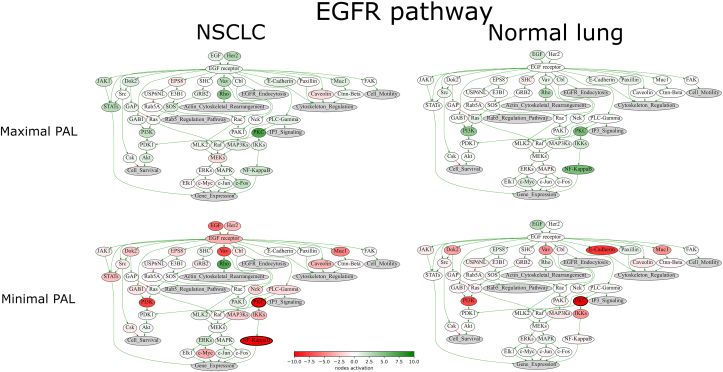
EGFR pathway (*Qiagen Pathway Central*) shown as an interacting network for samples with maximal (top) or minimal (bottom) PAL in colorectal cancer (left) vs normal colon (right). Green/red arrows indicate activation/inhibition interactions, respectively. The color depth of transcript nodes reflects the extent of node activation (logarithms of the expression fold change for each node, the reference is the geometric average between expression levels in all samples in the respective groups). Visualized using Oncobox software.

**Figure S4:**
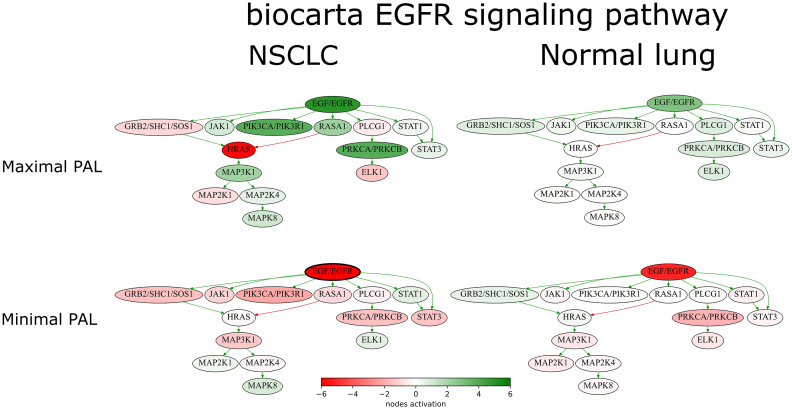
EGFR pathway (*Biocarta*) shown as an interacting network for samples with maximal (top) or minimal (bottom) PAL in colorectal cancer (left) vs normal colon (right). Green/red arrows indicate activation/inhibition interactions, respectively. The color depth of transcript nodes reflects the extent of node activation (logarithms of the expression fold change for each node, the reference is the geometric average between expression levels in all samples in the respective groups). Visualized using Oncobox software.

In both cancerous and normal tissues, there were samples with either strongly upregulated or downregulated EGFR pathways (Figure 2). Interestingly, in the samples with maximal or minimal PAL values, there were mostly similar patterns of up- or downregulated pathway nodes, respectively, between the normal and cancerous samples, for both versions of the EGFR pathway under investigation (Figures S1-S4). Taken together, these findings suggest that (*i*) there is a high level of EGFR pathway activation heterogeneity in both cancerous and normal tissues; (*ii*) in cancers, this heterogeneity cannot, for the most part, be explained by their intrinsic properties like the presence of clinically relevant mutations of EGFR pathway components.

Therefore, we hypothesized that the above EGFR pathway activation heterogeneity may be due to the “external” factors such as the presence of EGFR ligands in blood. To test this hypothesis, we analyzed the effects of peripheral human blood sera obtained from seven healthy donors, on the growth and sensitivity to EGFR-targeted drugs of squamous carcinoma cell line A431.

### 2.2 Human blood serum differentially affects colony formation by A431 cells

Tumor response to targeted therapy can be affected by various specific molecular factors present in the patient's body, like proteins and metabolites. They can affect the anti-proliferative action of the EGFR-targeted drugs both *in vivo* and *in vitro*. In this study, we measured the impacts of individual human peripheral blood serum samples on the cell colony formation in the presence of EGFR-targeted drugs.

**Figure 3:**
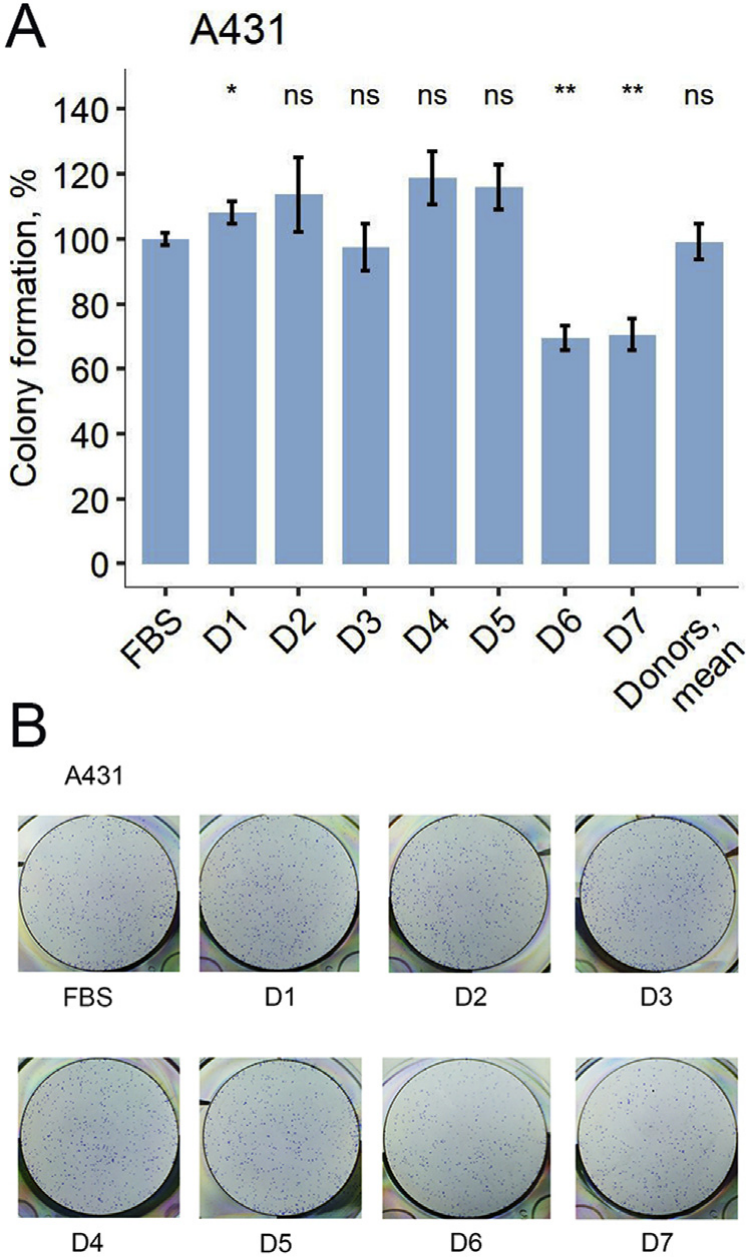
Colony formation by A431 cells in the growth media containing 10% FBS supplemented with human blood serum samples. (A) Colony formation is normalized to the FBS-only growth media (column FBS). The average of seven human donor serum samples is indicated as « mean » Asterisks indicate the significance of the difference from FBS-only experimental results. (B) Representative photographs of stained clonogenicity assay results were used for colony number calculations.

First, we studied the influence of human blood serum, in the absence of drugs, on the colony formation by cell line A431 that was obtained from 85 years old patient with epidermoid squamous cell carcinoma (Figure 3). This cell line was selected because it is a classical model for studying effects of EGFR-targeted drugs, including erlotinib and cetuximab, on the EGFR-overexpressing cancer cells [37, 38, 39]. We used human peripheral blood serum specimens obtained from seven healthy female donors. Female donor samples were used because the cell culture A431 originates from a female patient [40]. This could help avoiding possible gender-specific bias in the interactions between the cell culture model and human blood serum samples.

For all the samples tested, the growth media contained heat-inactivated fetal bovine serum (FBS). FBS concentration of 10% was enough to support maximum cell growth. We therefore tested cell colony formation ability in the growth medium containing 10% heat-inactivated FBS and supplemented with 2.5% human serum (or with 2.5% of additional heat-inactivated FBS for the controls).

It is important to note that among these seven serum samples tested we detected statistically significant variation, by nearly 50%, in the colony formation capabilities (Figure 3). We found that A431 colony formation was not affected by the majority of serum samples, and one serum sample (D1) could increase colony formation by about 10% (p < 0.05) (Figure 3). In turn, two individual serum samples (D6, D7) statistically significantly inhibited colony formation by ~30% (p < 0.01) (Figure 3).

### 2.3 Human blood serum samples differentially antagonize effects of EGFR-specific drugs on A431 colony formation

**Figure S5:**
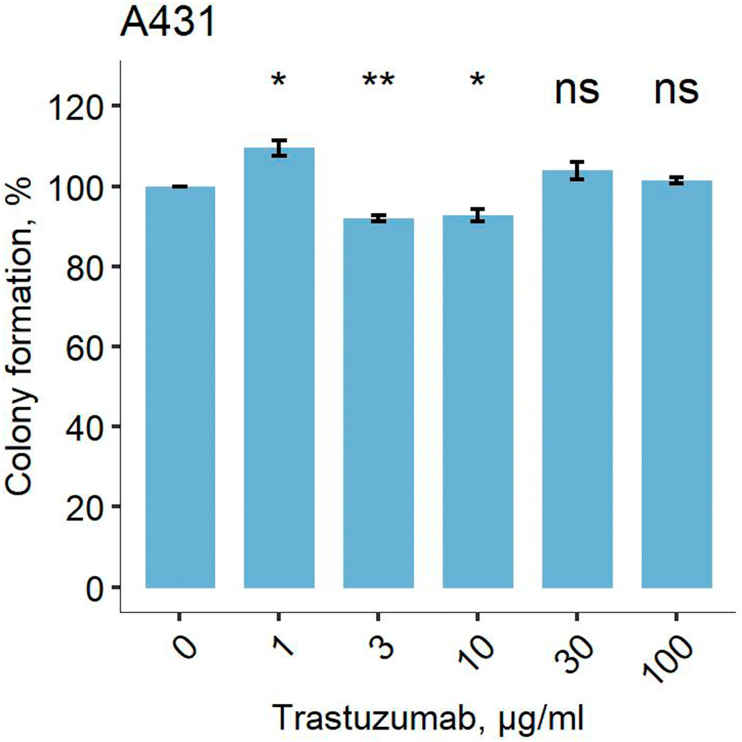
Colony formation by A431 cells in the presence of trastuzumab compared with the no-drug mode. Colony formation is given as a percentage of the number of colonies formed in the no-drug mode. The data are shown as the mean of three independent replicates ± standard deviation.

Epidermoid squamous carcinoma cell line A431 is known to express, on average, more EGFR than other cell lines of similar origin (~2 × 10^6^ EGFR molecules per cell versus ~4 × 10^4^ of HER2), and is highly sensitive to EGFR-targeted drugs [21, 41], but not sensitive to HER2-specific drug trastuzumab [42], Figure S5.

We then measured effects on A431 cells colony formation of the human donor blood sera and of two EGFR-specific targeted drugs: small molecular inhibitor erlotinib and therapeutic antibody cetuximab. Colony formation rate was normalized to the absence of drugs when only fetal bovine serum was present in the culture medium.

**Figure 4:**
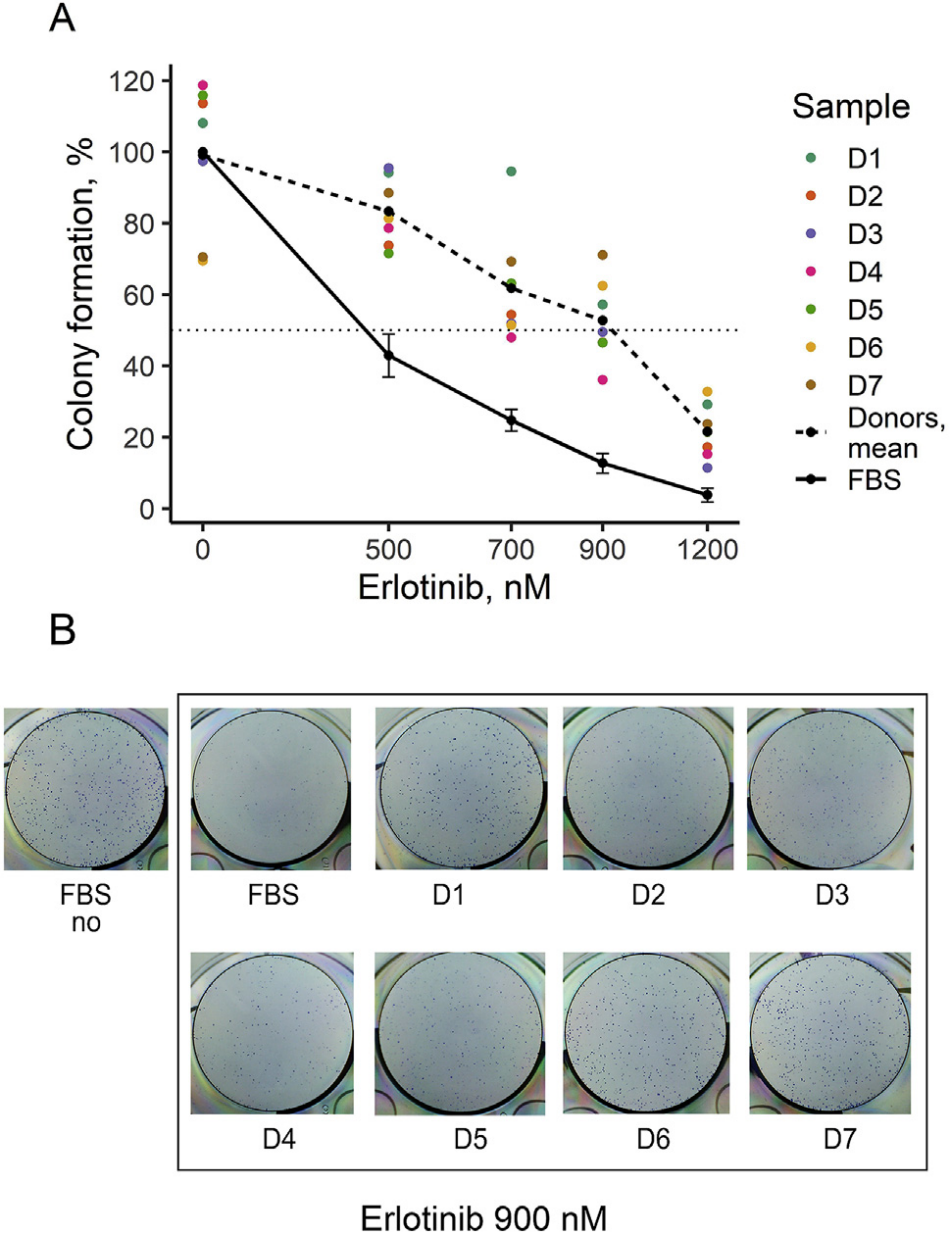
(A) A431 relative clonogenicity after treatment with erlotinib in the presence of human donor serum samples. Colored points represent average clonogenicity for each donor sample calculated from three replicates, D1-7 denotes the donor ID. The solid line (FBS) defines clonogenicity in the presence of FBS only; dotted line (mean) defines clonogenicity averaged for seven human donor samples. Colony formation is normalized to the results of the experiments with FBS-only growth media. (B) Representative photographs of stained clonogenicity assay wells, which were used for colony number calculations for 900 nM erlotinib - treated cells.

When only FBS was present, erlotinib IC50 was ~450 nM. We found that the human sera taken at a concentration as low as 2.5% could rescue cells from growth inhibition by erlotinib (Figure 4). For example, at 500 nM erlotinib (corresponds to its physiological concentration under standard therapeutic regimens [43, 44]), the colony formation ability was close to the level without the addition of drug. At erlotinib concentration of as high as 1200 nM, human sera increased A431 cell clonogenicity by approximately six times (~4% versus ~23%) on average. Human blood serum from each of the seven donors tested was able to abrogate the effect of erlotinib on A431 colony formation (Figure 4).

**Figure 5:**
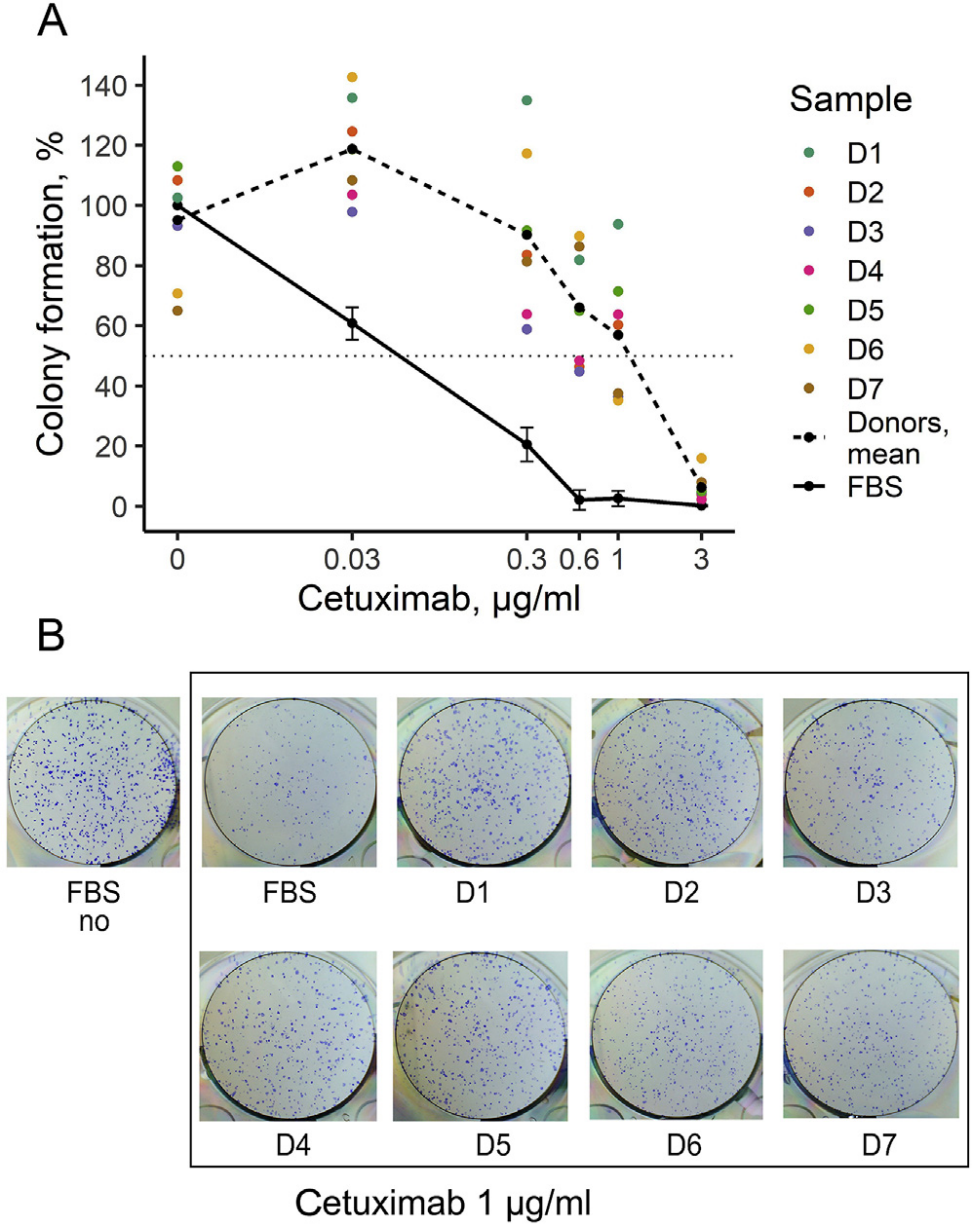
(A) A431 relative clonogenicity after treatment with cetuximab in the presence of human donor serum samples. Colored points represent average clonogenicity for each donor sample calculated from three replicates, D1-7 denotes the donor ID. The solid line (FBS) defines clonogenicity in the presence of FBS only; dotted line (mean) defines clonogenicity averaged for seven human donor samples. Colony formation is normalized to the results of the experiments with FBS-only growth media. (B). Representative photographs of stained clonogenicity assay wells, which were used for colony number calculations for 1 μg/ml cetuximab - treated cells.

We then similarly measured the effects of cetuximab on the colony formation, both separately and in combination with human peripheral blood serum (Figure 5). In the absence of human serum, cetuximab IC50 was ~0.06 μg/ml. As before, 2.5% human sera strongly antagonized cetuximab effects. For 0.3 μg/ml cetuximab, in five out of seven human sera tested, the colony formation ability was similar to that without the addition of the drug. With the other two serum samples, colony formation rate was about 60%, while in the absence of human serum the value was only ~20%. At 1 μg/ml cetuximab, colony formation rate varied between approximately 40% and 100% in the presence of human serum, while only a few colonies could be detected when no human serum was added. Finally, no colonies were detected at 3 μg/ml cetuximab without human serum, while colony formation rate was still above 10% in the presence of any of the human serum samples tested (Figure 5).

Comparison of cetuximab and erlotinib effects also showed that the impact of human sera was stronger in the case of the cetuximab treatment (compare Figures 4 and 5). In the presence of 2.5% human serum, IC50 of cetuximab was ~1.2 μg/ml (averaged over all serum samples), which is ~20 times higher than if only FBS was present (~0.06 μg/ml). For erlotinib, IC50 was ~450 nM if only FBS was present and ~900 nM, i.e. approximately twice as high in the presence of 2.5% human serum (averaged over all serum samples, Figure 4).

For both drugs tested, we observed variation of up to 100% in colony formation between the serum samples of seven different individuals (Figures 4 and 5). We performed the Kruskal-Wallis test to estimate how significant differences between serum samples are [45]. It is a non-parametric test to assess whether two or more groups of samples originate from the same distribution. A significant result (*p*-*value* less than 0.05) indicates here that at least one donor's sample is significantly different from the others. In our experiments, the Kruskal-Wallis test p-value was less than 0.01 at every drug concentration for both erlotinib and cetuximab (Tables 2 and 3). It confirms significant individual variations of human blood effects on EGFR pathway activation and rescuing cells from the growth inhibition by targeted drugs.

**Table 2:** Colony formation of A431 cells in the presence of erlotinib and 2.5% human serum (samples D1 – D7). The number of colonies in each experiment is normalized to the number of colonies formed in the absence of erlotinib and human serum. The data are given as the mean ± standard deviation. The Kruskal-Wallis rank-sum test was performed to estimate the significance of differences between the donors' sera.

Sample	Erlotinib, nM				
	0	500	700	900	1200
D1	108.1 ± 3.4	94.2 ± 2.9	94.6 ± 2	57.2 ± 3.9	29.2 ± 4.8
D2	113.6 ± 11.4	73.7 ± 4.6	54.4 ± 0.8	46.5 ± 3.8	17.2 ± 3.4
D3	97.4 ± 7.3	95.5 ± 5.7	51.9 ± 7.4	49.5 ± 4.5	11.4 ± 1.2
D4	118.7 ± 8.2	78.6 ± 2.8	48 ± 4.8	36.1 ± 4.6	15.2 ± 3.4
D5	115.9 ± 6.8	71.5 ± 1.3	63.1 ± 0.8	46.5 ± 3.7	21.4 ± 4.8
D6	69.5 ± 3.8	81.4 ± 1.7	51.4 ± 4.4	62.5 ± 4.3	32.8 ± 5.2
D7	70.5 ± 4.9	88.5 ± 1.4	69.3 ± 1.2	71.1 ± 2.8	23.7 ± 3.2
Donors, mean	99.1 ± 5.5	83.3 ± 2.1	61.8 ± 2	52.8 ± 2.1	21.6 ± 3.2
FBS	100 ± 1.9	42.9 ± 6	24.8 ± 3	12.7 ± 2.8	3.8 ± 2
Kruskal-Wallis test *p*-value	0.0053	0.0036	0.0045	0.0042	0.0082

**Table 3:** Colony formation of A431 cells in the presence of cetuximab and 2.5% human serum (samples D1 – D7). The number of colonies in each experiment is normalized to the number of colonies formed in the absence of cetuximab and human serum. The data are given as the mean ± standard deviation. The Kruskal-Wallis rank-sum test was performed to estimate the significance of differences between the donors' sera.

Sample	Cetuximab, μg/ml				
	0	0.1	0.3	1	3
D1	102.6 ± 6.8	135.8 ± 3.7	135 ± 4.3	81.9 ± 4.2	93.8 ± 4
D2	108.4 ± 6.7	124.6 ± 1.2	83.6 ± 3.4	46.3 ± 3.8	60.3 ± 2.2
D3	93.2 ± 7.1	97.9 ± 4.1	58.9 ± 5.4	44.7 ± 1.7	36.4 ± 1
D4	113.1 ± 0.5	103.6 ± 3.4	63.8 ± 7.8	48.5 ± 4.7	63.8 ± 2.9
D5	113 ± 5.7	118.7 ± 2.6	91.7 ± 3.7	64.9 ± 4.7	71.5 ± 4.7
D6	70.7 ± 7.8	142.7 ± 4.7	117.3 ± 7.1	89.8 ± 2.7	35.2 ± 3.2
D7	65 ± 7.1	108.4 ± 1.8	81.3 ± 5.4	86.3 ± 3.6	37.6 ± 3.2
Donors, mean	95.1 ± 0.8	118.8 ± 1	90.2 ± 2.3	66.1 ± 1.4	56.9 ± 1.8
FBS	100 ± 2	60.8 ± 5.4	20.6 ± 5.7	2.1 ± 3.3	2.6 ± 2.6
Kruskal-Wallis test *p*-value	0.0052	0.0024	0.0025	0.0036	0.0032

To further investigate whether the serum provides additive or antagonistic effects to the drugs tested, we measured *Bliss synergy score* (BS) with the discriminating threshold of BS > 5 for the synergistic effect, and BS < -5 for the antagonistic effect [36], of all the individual serum samples tested (Table 4). We found that the effects observed were essentially antagonistic to erlotinib and cetuximab activities in all the samples tested.

**Table 4:** Bliss synergy scores for serum samples obtained from different donors.

Donor	Erlotinib + serum Bliss synergy score	Cetuximab + serum Bliss synergy score
D1	-46	-72.4
D2	-24.05	-45.03
D3	-31.55	-32.39
D4	-19.48	-36.85
D5	-26.24	-50.82
D6	-42.37	-67.97
D7	-48.31	-53.08

**Figure 6:**
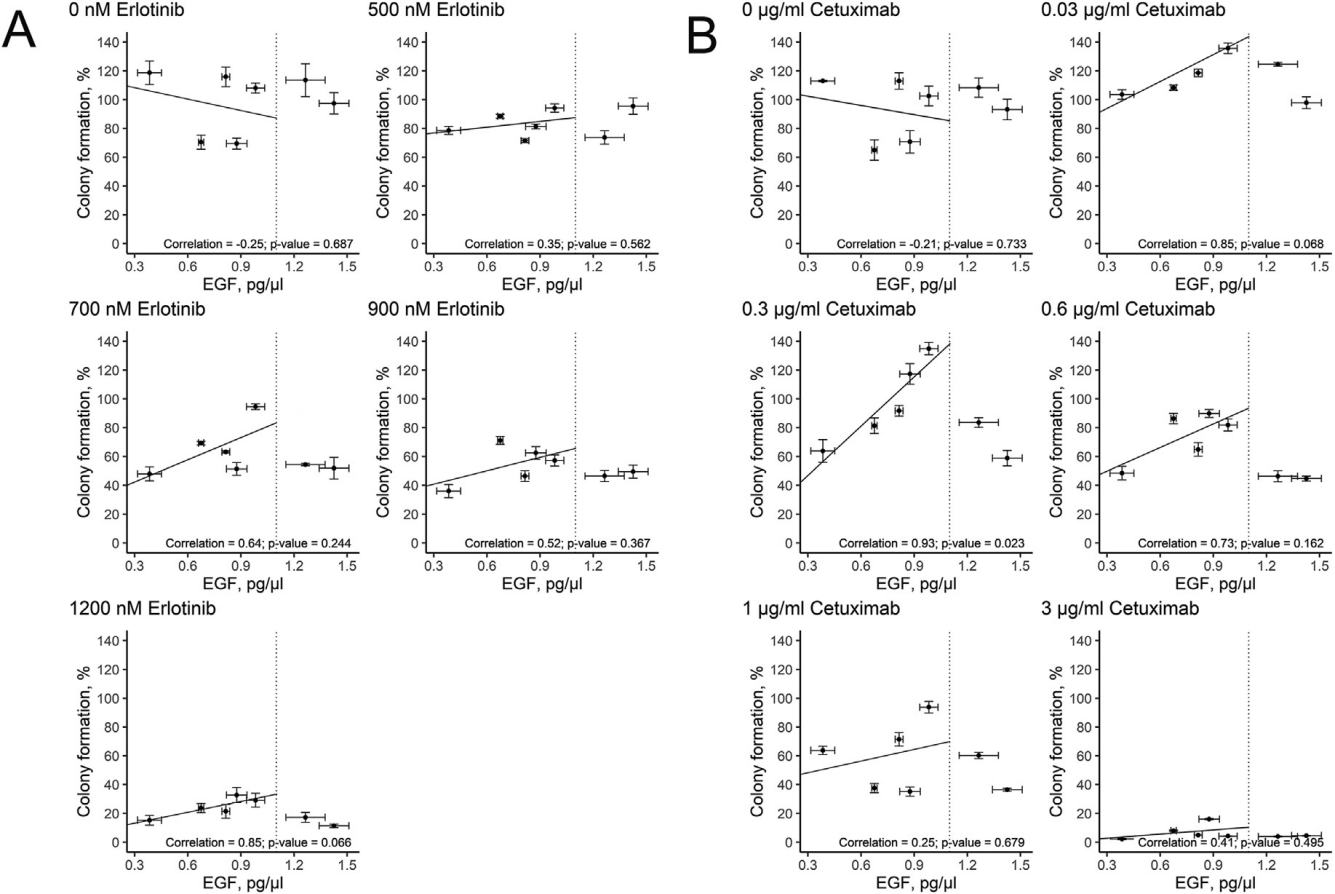
Colony formation efficacy by A431 cells treated with erlotinib (A) or cetuximab (B) are plotted against EGF concentration for each of seven human donor serum samples. Correlation between two values as well as a resulted trend (solid line) for the sera with moderate EGF concentration is indicated. The data are shown as the mean of three independent replicates ±standard deviation. The vertical line represents the 1.1 pg/μl threshold.

**Figure S6:**
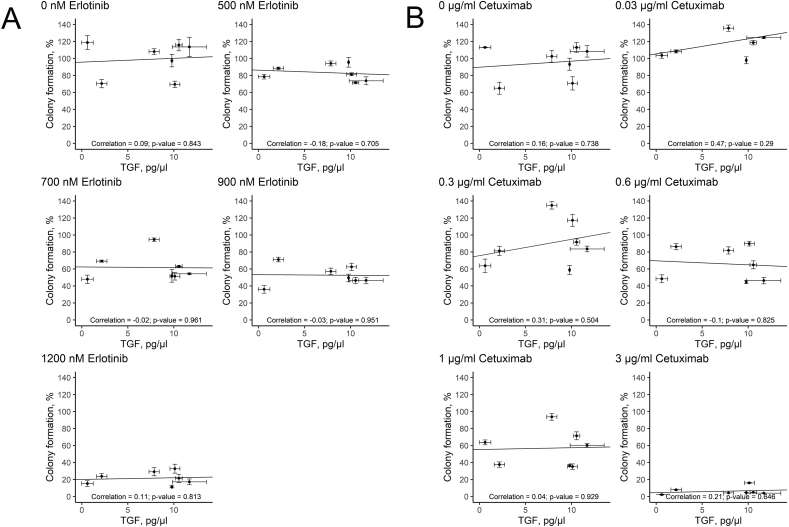
Dependence of colony formation efficacy by A431 cells treated with erlotinib (A) or cetuximab (B) on the presence of human donor serum samples, and concentration of TGFa in the respective serum samples. The data are shown as the mean of three independent replicates ± standard deviation.

We then assessed whether the colony formation capabilities were associated with the serum concentrations of EGFR ligands epidermal growth factor (EGF), transforming growth factor alpha (TGFa), and neuregulin (NRG), see Table 5. We found no detectable NRG concentrations in all seven serum samples under investigation and also found no correlation between colony formation capabilities and TGFa concentration in serum, both with and without drug added (Figure S6). However, for the sera with moderate concentrations of EGF (less than 1 pg/μl), we observed a trend of EGF concentration being congruent with the colony formation (Figure 6) in the presence of both targeted drugs. The trend was consistent in all the observations and was statistically significant at the concentration of 0.3 μg/ml of cetuximab added to the medium (correlation 0.93, *p* = 0.023; Figure 6B). Thus, at least several cell growth-promoting effects of human serum may be linked to the concentration of EGF, a major EGFR ligand.

**Table 5:** Serum concentration of EGFR ligands epidermal growth factor (EGF), transforming growth factor alpha (TGFa), and neuregulin (NRG), in pg/ml. Results of three independent ELISA measurements are given as the mean ± standard deviation.

	EGF	TGFa	NRG
D1	983 ± 61	7,9 ± 0,52	<50
D2	1265 ± 78	11,7 ± 0,71	<50
D3	1425 ± 87	9,8 ± 0,61	<50
D4	384 ± 23	0,6 ± 0,03	<50
D5	815 ± 55	10,5 ± 0,66	<50
D6	876 ± 53	10,1 ± 0,65	<50
D7	675 ± 43	2,2 ± 0,16	<50
D (-lig)∗	114 ± 3.8	4,2 ± 0,29	<50

∗ pooled serum devoid of EGFR ligands.

### 2.4 Serum devoid of EGFR ligands has decreased capacity for rescuing A431 cells from erlotinib

**Figure 7:**
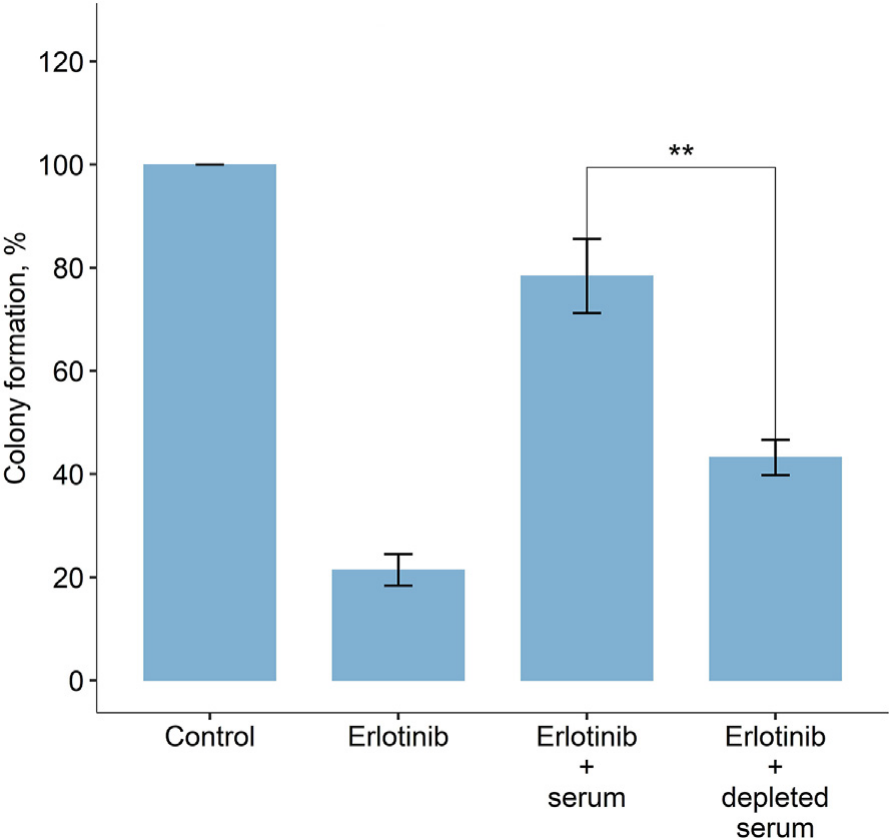
The effect of human serum devoid of EGFR ligands on the relative clonogenicity of A431 cells in the presence of erlotinib. *Control* means no serum and no drug added. *Erlotinib* means 700 nM erlotinib added. *Serum* means 5% of intact pooled human serum from seven donors added. *Depleted serum* means protein level-adjusted equivalent amount of pooled serum passed through the EGFR-immobilized affinity column. The data are shown as the mean of three independent replicates ±standard deviation.

It was shown previously for A431 cells that EGF, the most abundant EGFR ligand molecule in the blood, antagonizes cetuximab effects on cell growth [11]. We, therefore, investigated if the observed effect of human serum on colony formation inhibition persists when EGFR ligands are removed from the serum. For this purpose, we assembled an affinity column with agarose coated by EGFR extracellular domain (ectodomain; amino acids 1–601). We then obtained serum devoid of EGFR ligands by having the pooled serum from all seven donors pass through the affinity column and performed colony formation assay with both intact and EGFR ligands-depleted human serum. EGF concentration in depleted serum was 114 ± 3.8 pg/ml, which was approximately 8-fold lower than in the initial pooled serum. We used the amount of serum devoid of EGFR ligands adjusted to the same protein concentration as in the 5% pooled serum from all donors. We found that human serum devoid of EGFR ligands could antagonize the inhibitory effects of erlotinib to statistically significantly (*p* < 0.01) lesser extent, approximately two times lower compared with the intact serum + erlotinib (Figure 7). Thus, we conclude that EGF accounts for at least a significant part of drug-modulating capacities of human serum.

### 2.5 EGF can inhibit A431 cell growth at physiological concentrations and rescues A431 cells from growth inhibition by EGFR-targeted drugs

Conversely, we then explored the effects of recombinant human EGF on colony formation capability of A431 cells in the presence of EGFR-targeted drugs. While a patient is treated with anti-EGFR therapy, the drugs also interfere with endogenous EGFR ligands of the human body. We investigated the competitive effects of EGFR ligand EGF and EGFR inhibitory drugs on A431 colony formation capability. EGF is known to exert a dual action on cell culture growth by stimulating the cell proliferation at low, ~0.1 ng/ml, concentrations, or by inhibiting proliferation at concentrations of 70 ng/ml and higher in human MDA MB-468 and A431 cell lines [46]. In turn, EGF concentration in human blood can vary widely among individuals, with the reference range of ~0.3–1.7 ng/ml [47].

**Figure 8:**
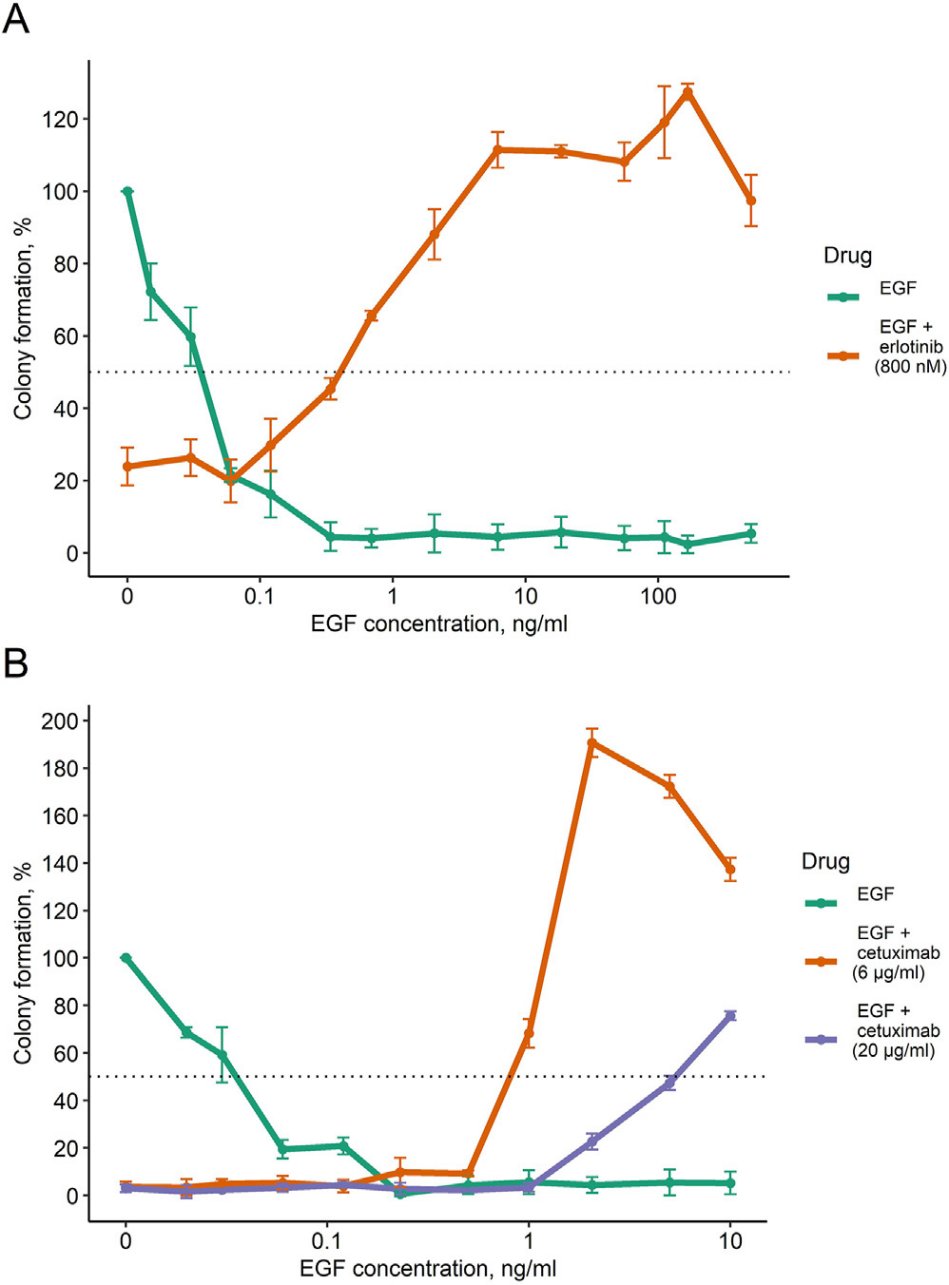
The effect of EGF on A431 clonogenicity in the presence of erlotinib (A) or cetuximab (B). Colony formation is given as a percentage of the number of colonies in the absence of EGF and drugs. The data are shown as the mean of three independent replicates ±standard deviation (see Tables 2 and 3).

We found that squamous carcinoma A431 cells could not form colonies at EGF concentrations exceeding 0.5 ng/ml (Figure 8). Thus, physiological levels of EGF could be sufficient to inhibit the proliferation of A431 cells. The observed EGF inhibitory effect on the cell colony formation was reversible: when the EGF-containing medium was replaced with the EGF-free medium after 8 or 24 h of incubation, the colony formation capability was restored (data not shown).

In agreement with the previous reports [11, 44], we found that combined treatment by the EGFR inhibitors and EGF restored colony formation in A431 cells (Figure 8). It could be due to the fine interplay of the anti- and pro- EGFR activating mechanisms [48]. Thus, we checked the dose-response effect of EGF in combination with drugs targeting EGFR. A431 cells could not form colonies in the presence of 0.5–500 ng/ml EGF (Figure 8A). In A431 cells, we detected ~450 nM IC50 for erlotinib (Figure 4). However, in the presence of EGF and 800 nM erlotinib, the colony formation capability could be restored completely, to a no drug – no ligand level, and even become higher (Figure 8A). Conversely, when EGF was present in the growth media at concentrations of 0.5 ng/ml and higher, the colony formation capability could be restored completely by adding therapeutic doses of erlotinib.

**Figure 9:**
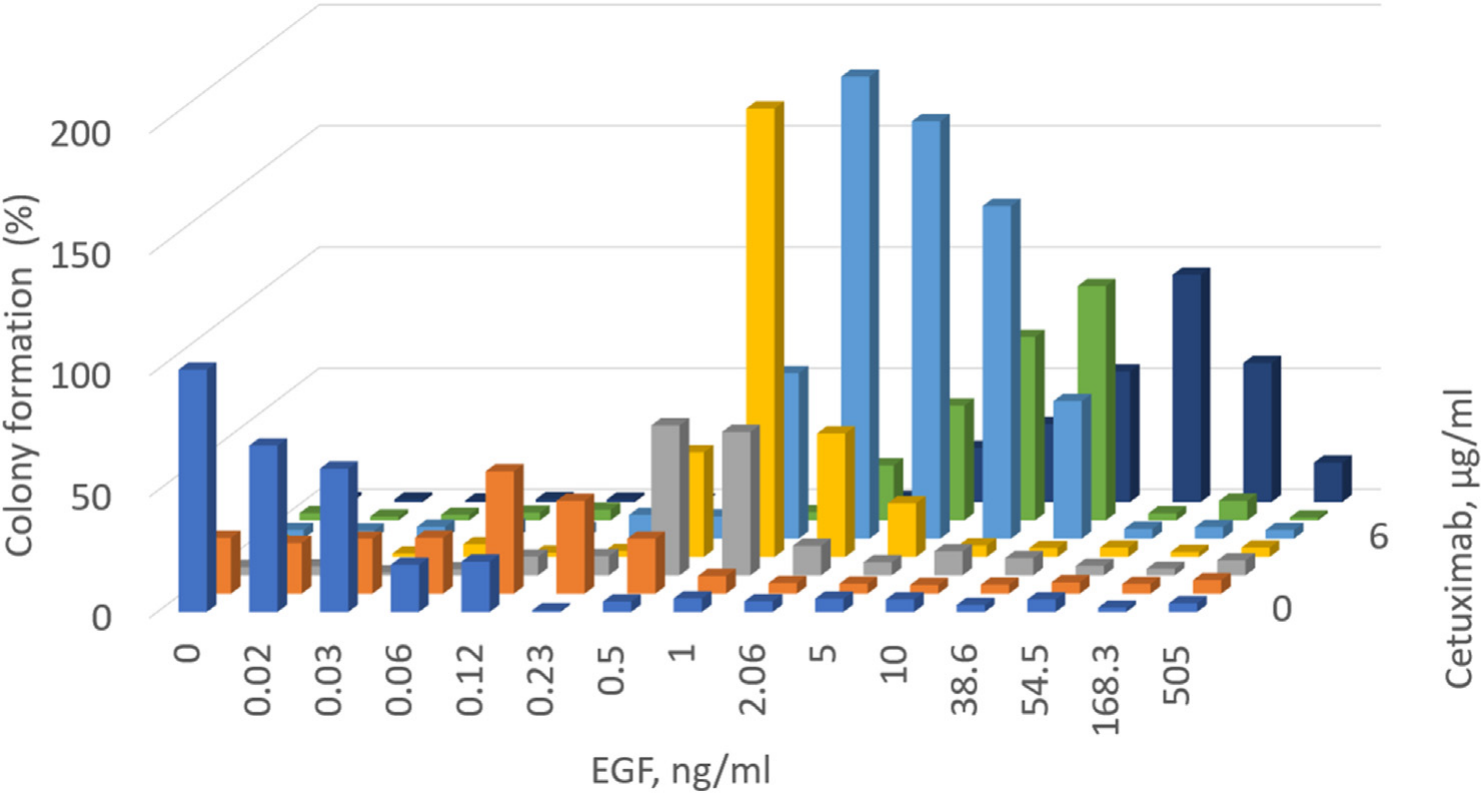
3D histogram for the dependence of A431 clonogenicity on the concentrations of cetuximab and EGF. The number of colonies in every experiment is normalized to the number of colonies in the absence of EGF and cetuximab. The experiments for each concentration point were performed with at least three biological replicates.

We observed similar effects with cetuximab. The results of dose-dependent colony formation assay for A431 cells at a wide range of cetuximab (0–100 μg/ml) and EGF (0–500 ng/ml) concentrations are shown in Figure 9. In our experiments, the cetuximab IC50 was ~0.06 μg/ml for A431 cells (Figure 5). However, at cetuximab concentrations of 6 and 20 μg/ml, the no drug level of colony formation was restored in the presence of 1–10 ng/ml EGF (Figure 8B); moreover, 50 ng/ml EGF restored colony formation even at cetuximab concentration of 100 μg/ml (Figure 9). For example, we observed that for 1 ng/ml EGF, which is close to its physiological concentration in peripheral blood, cetuximab IC50 was ~3 μg/ml, i.e. 60-fold higher than cetuximab IC50 in the absence of EGF. Furthermore, at 1 ng/ml EGF and 2 μg/ml cetuximab, and at 2 ng/ml EGF and 2–6 μg/ml cetuximab, the colony formation capability exceeded the basic (no-drug, no-EGF) level by approximately two times. Thus, our data confirmed that both EGFR inhibitors and EGFR ligands can inhibit the growth of squamous carcinoma cell colonies, and together they can counterbalance each other and enhance proliferation instead (Figure 9).

## 3 Discussion

The prediction of individual patient response to drug therapy is a challenging task in modern oncology. Most frequently, it involves genetic, epigenetic, or transcriptomic markers of individual tumors [18, 19, 49, 50, 51, 52, 53]. However, not only “intrinsic” tumor molecular properties can be relevant. Effects of blood serum/plasma components were considered in several clinical studies [15, 54]. Elucidation of underlying molecular mechanisms requires more detailed research involving cell culture models [20, 55, 56] and molecular pathway analysis [32, 57, 58].

Using human squamous carcinoma cell line A431, we show the impressive modulation of activities of two EGFR-specific drugs by human blood serum. Our results clearly demonstrate that conclusion about antitumor drug inhibitory effects could be erroneous, unless a patient-specific context, such as the concentration of EGFR ligands in individual blood serum, is taken into account. We show here that human serum has a strong potential to alter effective concentrations of EGFR-targeted drugs, and that these effects can vary statistically significantly by up to two times across individuals. For the first time, we show that human sera can rescue squamous carcinoma cells from growth suppression mediated by EGFR-targeted drugs. Thus, considering the patient-specific serum context could help personalize drug concentration adjustments to increase the efficacy of treatment and reduce its side effects. We found that human sera of seven healthy individuals had variable effects on the drug activities (up to 100% differences for both drugs investigated) and that this variation was statistically significant, as evidenced by the Kruskal-Wallis rank-sum test results.

In our experiments, the rescue effect of donor serum on A431 colony formation was lower in the case of cetuximab compared to erlotinib. This is not due to the lower half-life of cetuximab in serum/culture medium. Monoclonal antibodies including cetuximab are quite stable in cultivation media: light or incubation at 50 °C could decrease cetuximab activity, while its activity decreases slowly at 40 °C without shaking [59]. Erlotinib can be more stable as it presented recovery higher than 80% after incubation in water at 25 °C for more than one month [60].

However, when extrapolating these in vitro results on EGF ligand-EGFR chemotherapeutic drugs interplay in patients, one has to consider that the half-life of both cetuximab and erlotinib could be different in cell culture medium compared to the patient serum when injected. Cetuximab half-life in human body is 5–7 days [61, 62, 63], whereas for erlotinib it is nearly 1.5 days [64, 65]. To compensate this decay, cetuximab is injected weakly [66], while erlotinib is administrated daily [67].

Finally, we show that the proliferation of A431 squamous carcinoma cells is inhibited by relatively low, physiological doses of EGF. As EGF is essential for wound healing and tissue repair, our findings also suggest that for several weeks following the tumor removal surgery the remaining tumor could be under the control of endogenous EGF. Thus, our model predicts that administering EGFR-targeted drugs soon after tumor removal surgery can instead stimulate cancer cells to proliferate and to form new colonies in cooperation with internal molecules of EGF, which level may be increased following surgery. Thus, the individual EGF blood level can serve as an important biomarker to guide dosage aspects, timing, and overall design of cancer treatment involving EGFR-targeted drugs. It also provides an experimental *in vitro* model for measuring personalized context in predicting the efficiency of an anti-EGFR targeted treatment.

## 4 Materials and methods

### 4.1 Cell culture

The squamous carcinoma cell line A431 (ATCC CRL-1555) was taken from the collection of the Institute of Cytology, Russia. A431 cells were cultured at 37 °C and 5% CO_2_ in Dulbecco's Modified Eagle's Medium (DMEM; Gibco BRL) supplemented with 10–15% FBS (Gibco BRL) and 2 mM L-glutamin, 4.5 g/L glucose, 1% penicillin-streptomycin (Gibco BRL).

### 4.2 Clonogenicity assay (colony formation assay)

Trypsinized cells were plated in 25 cm^2^ flasks, 300–900 cells per flask, depending on the cell line. Alternatively, cells were plated on six-well culture plates with cell amounts optimized for experimental conditions (100–1000 cells). Plates with equal number of cells seeded were incubated for 24h–48h before treatment by recombinant hEGFR ligands or anti-EGFR drugs, or human sera. Downstream 4–18 days incubation of cells led to the formation of colonies, defined as more than 50 cells. The medium was discarded, and the cells were fixed using 4% formaldehyde for 10 min. Formaldehyde was then removed, and the cells were stained using 0.5% of crystal violet in 60% methanol and 1xPBS for 15 min and washed with water three times. Colonies were detected and counted by openCFU software [68] (with regularity parameter set to 0). Colony formation (CF) was calculated as a ratio of the number of colonies formed by treated and untreated cells. All experiments were conducted with at least three independent replicates.

### 4.3 EGFR ligands depletion from human blood serum

Expression vector sEGFR621 encoding for ectodomain of EGFR (amino acids 1–621) fused with the FLAG peptide was kindly provided by Dr. Tim Adams (CSIRO Manufacturing, VIC, Australia). HEK 293 cells at 50% confluence were transfected with the vector using Lipofectamine 3000 transfection Kit (Invitrogen) according to the manufacturer's manual. The cultivation medium was collected 6 h following transfection, and then a fresh medium was added to the cells for additional 16 h. Then the medium was collected and replaced with a fresh medium for 24 more hours. Cultivation media were pooled and passed through the anti-FLAG agarose column as a batch procedure, as per the manufacturer's instructions (Sigma-Aldrich). The pooled human serum sample was obtained by mixing the same volumes of sera from all the donors included in this investigation. The pooled sample was passed through the column with immobilized EGFR ectodomain, and then this procedure was repeated so that the serum contacted the column for 20 min.

### 4.4 EGFR-targeted drugs, EGF, and human serum samples

Cetuximab (Erbitux), solution 5 mg/ml, was purchased from Merck, stored at 4 °C; erlotinib, dry powder, was purchased from Sigma-Aldrich, stored at -20 °C as 10 mM solution in DMSO. EGF, dry powder, was purchased from PanEco, stored at -20 °C.

Peripheral blood samples from seven unrelated healthy 23–64 years old female donors were collected in two 8-ml vacuette tubes containing pro-coagulant and gel (Greiner), and serum was prepared within 3–4 h upon blood collection: tubes were centrifuged at 2500 rpm for 15 min, sera were aliquoted and stored at -75 °C. For all the human biomaterials investigated, informed written consents to participate in this study and to communicate the results in the form of a scientific report were collected from the corresponding donors. The procedure of taking human materials, the consent procedure, and the design of this study were approved by the ethical committee of the Vitamed Clinical Center, Moscow.

### 4.5 Calculation of EGFR pathway activation

RNA sequencing gene expression profiles were extracted from GTEx and TCGA databases. COAD (colon adenocarcinoma) and READ (rectal adenocarcinoma) TCGA samples were pooled as the colorectal cancer group (n = 604). LUAD (lung adenocarcinoma) and LUSC (lung squamous cell carcinoma) TCGA samples were pooled as the non-small cell lung cancer group (NSCLC, n = 1026). These samplings included only the specimens with matched mutation data deduced using whole-exome sequencing profiles. The sample was considered mutated if at least one of the following mutations was found in TCGA vcf file (Mutect2 variant caller): *BRAF* codon 600; *NRAS* or *KRAS* codons 12, 13, 59, 61, 117, 146; *EGFR* codon 858 or deletion in exon 19. Normal samples originating from lung (n = 578) and colon (n = 779) were selected according to the GTEx database label in the SMTS field. Raw gene counts from all datasets were merged into a single matrix, and quantile normalized [69]. To minimize the effect of the group size on data variation, we then randomly selected 578 individual samples from each group (corresponds to the size of the minimal group under comparison) for further analyses and calculated pathway activation levels for each sample using Oncobox software [32].

Pathway activation level (PAL) characterizes cumulative changes in expression levels of genes belonging to a certain molecular pathway. PAL is calculated as follows:

$$PAL_{p}=\underset{n}{\sum }ARR_{np}*\frac{\mathrm{lg}\left(CNR_{n}\right)}{\sum_{n}\left|ARR_{np}\right|}$$

where PALp is PAL for pathway p, CNRn is case-to-normal ratio, the ratio of gene n expression level in a tumor sample under study to an average level in the control group; ARR (activator/repressor role) is a Boolean flag that depends on the function of gene n product in pathway p. ARR value is −1 if gene product n inhibits pathway p; 1 if n activates the pathway; 0 if n has ambiguous or unclear role in the pathway; 0.5 or −0.5, if n is more a pathway activator or its inhibitor, respectively.

### 4.6 Statistical analysis

T-test was used for pairwise comparisons. On the graphs, *p-*values are represented as follows: ∗∗∗∗ - less than 0.0001, ∗∗∗ - less than 0.001, ∗∗ - less than 0.01, ∗ - less than 0.05.

To quantify drug synergy, we calculated Bliss model scores using R Calculate Synergy function without baseline correction from Synergy Finder package [70]. This package utilizes the following model equation:

Y_BLISS_ = Y1 + Y2 – Y1∗Y2

where Y is the compound's effect (fractional inhibition of cell growth). We considered effect synergistic when the corresponding Y_BLISS_ score was greater than 5, antagonistic when it was lower than −5, and additive when otherwise [36].

## Declarations

### Author contribution statement

Dmitry Kamashev: Conceived and designed the experiments; Performed the experiments; Analyzed and interpreted the data; Contributed reagents, materials, analysis tools or data; Wrote the paper.

Maksim Sorokin: Analyzed and interpreted the data.

Irina Kochergina, Uliana Vladimirova, Nina Shaban: Performed the experiments.

Aleksey Drobyshev: Performed the experiments; Wrote the paper.

Marianna Zolotovskaia, Mikhail Raevskiy, Denis Kuzmin: Analyzed and interpreted the data; Wrote the paper.

Igor Vorotnikov: Conceived and designed the experiments; Performed the experiments.

Anton Buzdin: Conceived and designed the experiments; Analyzed and interpreted the data; Contributed reagents, materials, analysis tools or data; Wrote the paper.

### Funding statement

This study was supported by the Russian Science Foundation grant (18-15-00061).

### Data availability statement

Data included in article/supplementary material/referenced in article.

### Declaration of interests statement

The authors declare no conflict of interest.

### Additional information

No additional information is available for this paper.
